# Atypical antipsychotic use does not impact weight gain for individuals with extreme anorexia nervosa: a retrospective case–control study

**DOI:** 10.1186/s40337-023-00941-6

**Published:** 2023-12-06

**Authors:** Maryrose Bauschka, Ashlie Watters, Dan Blalock, Asma Farooq, Philip Mehler, Dennis Gibson

**Affiliations:** 1 ACUTE Center for Eating Disorders & Severe Malnutrition at Denver Health, 777 Bannock Street, Denver, CO 80204 USA; 2 Department of Medicine, University of Colorado School of Medicine, Aurora, CO USA; 3 Eating Recovery Center, Denver, CO USA; 4 University of Utah School of Medicine, Salt Lake City, UT USA; 5 Center of Innovation to Accelerate Discovery and Practice Transformation, Durham Veterans Affairs Medical Center, Durham, NC USA; 6 Department of Psychiatry and Behavioral Sciences, Duke University School of Medicine, Durham, NC USA

**Keywords:** Anorexia nervosa, Antipsychotics, Weight trend, Weight gain, Medications

## Abstract

**Background:**

There are no U.S. Food and Drug Administration (FDA)-approved medications for the treatment of anorexia nervosa (AN). Various medication classes have been evaluated for benefits in this population, including antipsychotics. Studies focused on use of antipsychotics for assistance with weight restoration in AN produced conflicting results. While current evidence does not suggest that antipsychotic medications can be generally recommended for persons with AN, some individuals might benefit from an antipsychotic medication for anxiety, mood, and the cognitive distortions that accompany the illness. It is well-established that atypical antipsychotics can cause weight gain when taken by other psychiatric populations. This published data can understandably limit the willingness of persons with AN to trial these medications. Given the conflicting results of studies examining antipsychotic-related weight gain in AN, it is currently hypothesized that individuals with extreme anorexia nervosa, restricting type, do not experience the weight gain seen in other psychiatric populations utilizing atypical antipsychotics.

**Methods:**

Two hundred seventy-six individuals with extreme AN were enrolled in this retrospective, case–control study between April 1, 2016 and June 30, 2022 utilizing study-specific inclusion and exclusion criteria. Clinical and demographic data, including use of atypical antipsychotics and weights, were retrospectively obtained from chart review. Variables were assessed for normality using univariate statistics. Continuous variables were described using means (M) and standard deviations (SD) or medians and interquartile ranges (IQR) based on normality. Differences in weight gain between cohorts was ascertained via independent samples t-test. P values of < 0.05 were considered statistically significant, and all analyses were completed using SAS Enterprise Guide software version 7.1 (SAS Institute, Cary, NC) and R version 4.3.1 (R Core Team, 2023).

**Results:**

Use of antipsychotics in this population of individuals with extreme AN did not impact the rate of weight gain (M: 1.7 kg/week, SD: 0.9 and 0.8, for cases and controls respectively).

**Conclusions:**

Weight gain is often cited by individuals with AN as a feared side effect of antipsychotic medications. In this study, there was no difference in weight trends for individuals taking atypical antipsychotic medications during the refeeding process compared with individuals who were not.

## Abstract (plain language summary)

While antipsychotics are not FDA-approved for, and cannot be generally recommended for, people diagnosed with anorexia nervosa, there are those who may derive benefit from utilizing these medications for their rigid and circular thoughts associated with the disorder, or for their mood and anxiety symptoms. Yet, many such individuals are hesitant to take these medications due to their fear of the weight gain which has been associated with their usage. In this study population of individuals suffering from extreme AN, who were undergoing refeeding and weight restoration, there was no impact on weight gain trends in individuals taking atypical antipsychotics compared to individuals who were not taking these medications.

## Background

While there are no FDA approved medications for anorexia nervosa (AN), psychopharmacological agents of a variety of classes are utilized to target comorbidities and eating disorder-related symptoms like anxiety and perseveration at mealtimes and rigid and distressing cognitions related to body image, weight or nutritional intake. Studies of selective serotonin reuptake inhibitors (SSRIs) in AN have not demonstrated significant differences in symptoms of psychopathology or depressive symptoms during the weight restoration phase of treatment [1–3]; there are no published data on selective norepinephrine reuptake inhibitors (SNRIs), and other classes of antidepressants such as tricyclic amines (TCAs) and bupropion are generally contraindicated given their side effect profile. Mirtazapine has shown some positive results [4–6]. However, it is crucial to note that tryptophan, which must be obtained from nutritional intake, is the precursor to serotonin. If nutritional intake is limited, serotonin production and the neurotransmitter system will be impacted. This may explain the poor responses seen to serotonergic medications in low-weighted individuals; moreover, it has been proposed that serotonin production may not fully normalize until an individual achieves 85–90% of their IBW [1, 7]. As such, in individuals with extreme AN, the utility of these medications is limited, necessitating exploration of other medication classes to treat psychiatric symptoms in this population.

Studies focused on the use of antipsychotics for assistance with weight restoration, with weight gain being the primary outcome of interest, have produced conflicting results complicated by very high drop-out rates, side effects and patient resistance to these medications [8–20]. Olanzapine and aripiprazole have shown the most promise, although most studies have focused on rate of weight gain or end-of-treatment body mass index (BMI), rather than addressing psychopathology [8, 9, 14, 15, 18–20]. A meta-analysis examining the reasons for dropout and the metabolic effects of atypical antipsychotics in AN found that personal reasons or factors, and not side effects or metabolic events, were the most common reasons cited for dropout [21]. Other meta-analyses in people with AN have not reported statistically significant differences in weight or changes in BMI between placebo groups and groups receiving antipsychotics [20, 22–24]. Despite these mixed findings in populations with AN, people suffering with AN are often reluctant to trial these medications, for their eating disorder or comorbidity-related psychopathology, given the widely published data identifying weight gain as a side effect in other psychiatric disorders, and based on the promulgated existent lore.

While the current evidence does not as yet support FDA approval for antipsychotic medications to be recommended for individuals with AN, it is possible some individuals or subgroups of this population might benefit from an antipsychotic medication for anxiety, mood, and the rigid and distorted thinking that accompanies the illness. These highly prevalent symptoms can add considerable distress to refeeding and negatively impact the weight restoration process. However, it is well-established that atypical antipsychotics can cause weight gain in other psychiatric populations [25–27], and this widely published data often limits the willingness of individuals with AN, who inherently fear weight gain, to trial these medications. Given the heretofore conflicting results of studies in AN, as to the impact of these medications on weight, and anecdotal promising results espoused by clinicians treating individuals with AN, it was hypothesized that individuals with extreme AN, restricting type (AN-R), would not experience the weight gain seen in other psychiatric populations who utilize atypical antipsychotics. The aim of this study was to evaluate if use of atypical antipsychotics targeting psychopathology alone had any inadvertent impact on weight gain in this patient population diagnosed with extreme AN-R.

## Methods

A total of 276 female individuals admitted to the ACUTE Center for Eating Disorders and Severe Malnutrition at Denver Health (ACUTE), a medical stabilization hospital-based unit dedicated to providing care for severely ill and medically-compromised eating-disordered individuals, were enrolled for this retrospective, case–control study between April 1, 2016 and June 30, 2022. This study was evaluated and approved by the Colorado Multiple Institutions Review Board. Both cases and controls were eligible for inclusion if they were over the age of 18 years old and if they were diagnosed with AN-R by ACUTE psychiatrists or psychologists based on DSM-5 criteria [28]. All consecutive eligible patients were included. Individuals were included in the case cohort if they were newly prescribed an atypical antipsychotic medication or were switched from one antipsychotic to a different one during their first week of admission to ACUTE. Medications included in the analysis included: olanzapine (n = 39), aripiprazole (n = 6), ziprasidone (n = 1), and quetiapine (n = 36). Conversely, individuals were excluded if they were already taking an antipsychotic medication on admission to ACUTE and this antipsychotic medication was continued. The control cohort included randomly selected individuals who were age-matched to the case cohort and if they were never prescribed an antipsychotic medication during their admission. Male individuals were excluded as the number of males was inadequate for comparison purposes. Individuals were also excluded from both cohorts if they were diagnosed with diabetes mellitus type 1 or type 2 or if their length of hospitalization on the ACUTE unit was less than one week. Patients with diabetes mellitus type 1 were excluded as glucose is often difficult to control in the early stages of refeeding these fragile patients, as their insulin requirements, notwithstanding their diabetes mellitus type 1, are small, and as their kilocalories (kcal) are increased this all can impact their weight trends. Patients with diabetes mellitus type 2, were excluded as commons medications utilized for this diagnosis, like metformin, can cause weight loss, and thiazolidinediones, can cause weight gain, which could confound results.

Clinical and demographic data, including medications, were retrospectively obtained from review of the medical record. Per the unit protocol, females are generally started on a 1400 kcal diet and their daily intake is increased by 300–400 kcals every 2–3 days to achieve 3–4 lbs of weight gain per week. Individuals are blinded to their daily weights. The amount of weight gain per week was calculated by taking the difference between the admission weight and the discharge weight, divided by the total number of days of hospitalization, then multiplied by seven.

Variables were assessed for normality using univariate statistics. Continuous variables were described using means (M) and standard deviations (SD) or medians and interquartile ranges (IQR) based on normality. Differences in weight gain between cohorts was ascertained via independent samples t-test. Multivariate linear regressions were used to examine differences in weight at discharge controlling for admission weight differences. P values of < 0.05 were considered statistically significant, and all analyses were completed using SAS Enterprise Guide software version 7.1 (SAS Institute, Cary, NC) and R version 4.3.1 (R Core Team, 2023).

## Results

Table 1 presents demographic and clinical information for the overall cohort, and for cases and controls. The majority of the cohort was White or Caucasian (90%), average age was 31.7 years (SD: 12.8), average admission BMI was 13.2 kg/m^2^ (SD: 2.0) and average %IBW on admission was 63.6% (SD: 9.7).

**Table 1:** Demographic and clinical information for cases and controls

	Total (n = 276)	Cases (n = 82)	Controls (n = 194)
	Mean (SD)	Mean (SD)	Mean (SD)
Age (yrs)	31.7 (12.8)	31.7 (12.7)	31.6 (12.9)
Admission BMI (kg/m^2^)	13.2 (2.0)	13.5 (2.0)	13.0 (2.0)
Admission %IBW	63.6 (9.7)	65.4 (9.7)	62.9 (9.6)
Discharge BMI (kg/m^2^)	15.3 (1.2)	15.6 (1.2)	15.2 (1.2)
Discharge %IBW	74.2 (5.9)	75.5 (6.2)	73.6 (5.7)
BMI Change (kg/m^2^)	2.17 (1.51)	2.08 (1.57)	2.21 (1.49)
%IBW Change	10.53 (7.40)	10.11 (7.73)	10.71 (7.27)
Kg per Week Gained	1.7 (0.8)	1.7 (0.9)	1.7 (0.8)
Length of Hospitalization (days)	25.9 (12.6)	24.4 (12.7)	26.5 (12.5)
Duration of Illness (yrs)*	13.7 (11.4)	13.9 (11.0)	13.6 (12.0)
Kcals on Discharge	3409 (598)	3312 (606)	3450 (591)
Aripiprazole (mg; n = 6)	3.42 (3.23)	3.42 (3.23)	n/a
Ziprasidone (mg; n = 1)	20.00 (0.0)	20.00 (0.0)	n/a
Quetiapine (mg; n = 36)	137.03 (175.38)	137.03 (175.38)	n/a
Olanzapine (mg; n = 39)	3.97 (3.50)	3.97 (3.50)	n/a

*doi avail for 69 cases/161 controls

Cases and controls did not differ significantly by age (t = 0.04, p = 0.97) or duration of illness (t = 0.17, p = 0.87). Cases had significantly higher admission BMI (t = 1.99, p = 0.048) and admission %IBW (t = 1.99, p = 0.048) than controls. All people were started on the same number of kcals per day on admission, and there was no significant difference in total daily kcals consumed by the time of discharge (t = −1.14, p = 0.26). Changes from admission to discharge in BMI (t = −0.61, p = 0.54) and %IBW (t = −0.59, p = 0.56) did not differ significantly between cases and controls. Weight gain in kilograms per week did not differ significantly between cases and controls (t = 0.74, p = 0.46). Finally, when accounting for admission BMI or admission %IBW, respectively, discharge BMI (b = −0.20, t = −1.69, p = 0.09) and discharge %IBW (b = −0.95, t = −1.59, p = 0.11) did not differ significantly between cases and controls.

## Discussion

In this study patient population of individuals with severe AN, the main finding was that there was no significant difference between those individuals who received antipsychotics starting in the first week of their admission and those who did not with respect to weight gain trend defined by kilograms gained per week.

These findings are of potential import because there are no FDA-approved medications for AN.

As previously noted, weight gain is a well-established side effect of atypical antipsychotics in other, non-AN, psychiatric populations [25–27], and there are a number of proposed highly complex neurobiological mechanisms to explain this phenomenon, generally mediated through binding to histamine, norepinephrine, dopamine and serotonin receptors. While a full discussion of the proposed mechanisms for antipsychotic-induced weight gain is beyond the scope of this paper, the degree of histamine H1 receptor affinity of the medication can predict weight gain. Antagonism at these receptors interferes with satiety signals from the gut and, along with serotonin receptor 2C antagonism, can stimulate appetite [29, 30]. Atypical antipsychotics appear to increase leptin levels, which impacts hunger cues and energy balance, and have been shown to result in insulin resistance [30–32]. In other psychiatric populations, identified risk factors for weight gain include younger age [33], female sex assigned at birth [34], non-white race [33], low baseline BMI [35], and certain pharmacogenetic profiles including the 5HT2C-759T allele [36] and MTHFR 677C/T “CC” genotype [37]. It is unclear why atypical antipsychotics do not appear to definitively impact weight gain or weight trends in extreme AN, particularly given that several of the identified risk factors in other populations like younger age, female sex assigned and birth and low BMI, are frequently also present in the individuals with anorexia nervosa. This is an area of potential future research.

Regarding clinical implications, this study extends knowledge regarding the relationship, if any, between antipsychotic use and weight gain in individuals with AN, and it provides a comparison to the relationship between these medications and weight changes seen in other psychiatric populations. Given the retrospective nature of the current study, data on changes in psychological scales is not available for the described patient population, and as previously noted, the use of these atypical antipsychotics in patients with AN does not have an evidence base at this time. For example, while Bissada et al., found a decrease in obsessions and compulsions as measured by the Yale Brown Obsessive–Compulsive Scale (Y-BOCs) and noted a greater increase in weight compared to placebo for day hospital patients receiving olanzapine, in the multicenter study published by Attia et al., there was no significant change identified in obsessionality, anxiety, weight concerns, restraint, or eating concerns in outpatients while noting a modest therapeutic effect on weight [8, 38]. While there is limited data to suggest that that atypical antipsychotics are associated with improved psychological symptoms, there still may be individual patients or subgroups of this population who still might benefit from an antipsychotic medication for anxiety, mood, and the rigid and distorted thinking that accompanies the illness. This study can offer some reassurance countering the fear of weight gain that individuals with AN often cite as a reason to avoid trying these medications, thereby encouraging potential expansion of the limited medication armamentarium for this difficult to treat population.

There are several limitations and strengths to this study. Firstly, this study is specific to individuals meeting criteria for extreme AN, individuals assigned female at birth, and adults only. The study population was 90% Caucasian, limiting generalizability to non-white individuals. There were a limited number of medications involved in the analysis, and the atypical antipsychotics included have variable associations with weight gain, for example, in other clinical populations aripiprazole and ziprasidone are considered weight neutral, while olanzapine is consistently associated with weight gain. Conclusions can only be drawn related to the generally low doses of the medications utilized in this population over a relatively short period of time, and these doses varied with respect to therapeutic equivalence. Long-term follow up data are not available given the limited length of stay on a medical stabilization unit that singularly specializes in medical stabilization of the complications of severe malnutrition secondary to eating disorders. And certainly, the retrospective nature of a case–control study is a limitation and impacts the ability of the authors to compare those of previously published randomized control trials. A strength is data specific to individuals with extreme AN on a medical stabilization unit, which is not a population specifically represented in prior studies and may also account for the difference in findings regarding weight gain compared to other studies in day hospital and outpatients with anorexia nervosa [8–20, 38].

## Conclusions

Accelerated weight gain, a side effect of atypical antipsychotics, is feared by individuals struggling with AN who might otherwise benefit from these medications. However, this retrospective case–control study in individuals with extreme AN-R on a medical stabilization unit, does not support the supposition that people with anorexia nervosa will experience accelerated weight gain during refeeding and weight restoration. Further controlled studies will be needed to extend and confirm, or refute, these findings regarding weight gain, and to assess the impact of atypical antipsychotics on psychological symptoms associated with the eating disorder itself or comorbid psychiatric diagnoses.

## Data Availability

The datasets used and/or analyzed during the current study are available from the corresponding author on reasonable request.

## Abbreviations

AN: Anorexia nervosa
AN-R: Anorexia nervosa, restricting type
kcal: Kilocalories
